# Contribution of spike timing to the neural code: from fast to slow timescales

**DOI:** 10.1007/s00422-026-01042-8

**Published:** 2026-04-14

**Authors:** Stefano Panzeri, Nicola Marie Engel, Marco Celotto

**Affiliations:** 1 Institute for Neural Information Processing, University Medical Center Hamburg-Eppendorf (UKE), 22527 Hamburg, Germany; 2 Picower Institute for Learning and Memory, Massachusetts Institute of Technology, Cambridge, MA 02139 USA

**Keywords:** Spike timing, Information coding, Sensory coding

## Abstract

The publication of Mainen and Sejnowski’s 1995 seminal paper strongly renewed interest in how spike timing contributes to the neural code. In the 3 decades since then, considerable experimental and theoretical research has investigated the timescales at which spike timing contributes to the neural code. Here we review theoretical and experimental research of the last 30 years aimed at defining conceptually and measuring operationally these timescales. By a critical review of the literature, we individuate six broad classes of timescales that have been conceptualized and operationalized: the maximal temporal precision of spiking that a neuron can achieve, the encoding time window (the time window containing the information-bearing spike times), the encoding timescale (the coarsest time resolution for measuring spikes without losing information), the maximal discrimination precision timescale (the smallest spike time difference that can be discriminated behaviorally), the encoding-readout intersection timescale (the maximal timing precision at which stimulus information encoded in neural activity is also actually read out to inform behavior), and the information consistency timescale (measuring the stability of information encoding over time). Together, this work has revealed short and long timescales that influence information coding and affect behavior. Short encoding timescales, from milliseconds to tens of milliseconds, are useful for sensory information encoding and perception. Long consistency timescales, ranging from hundreds of milliseconds to seconds, are useful for accumulating evidence and stabilizing decisions.

## Introduction

**Fig. 1:**
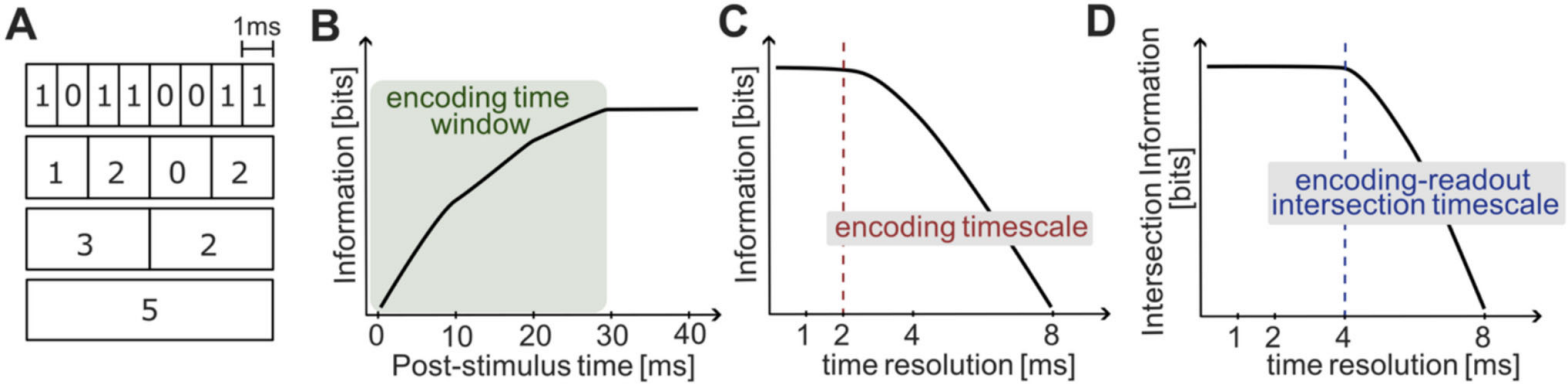
Spike timing resolution and operational measures of timescales. **A** Illustration of measuring spike time with increasing resolution, from a 1-ms precision spike timing (top) down to a spike rate code counting the spikes in the considered window. **B** Illustration with a cartoon of the operational computation of the encoding time window as the shortest time window that contains all stimulus information, evaluated as the first post-stimulus time (x-axis) at which the cumulative stimulus information in neural activity (y-axis) reaches the maximum value over time. **C** Illustration of the operational computation of the encoding timescale. The stimulus information (y-axis) is computed as a function of the time resolution (x-axis) and the encoding timescale is selected as the longest timescale (coarsest resolution) that gives the maximal information available in neural activity. **D** Illustration of the operational computation of the encoding-readout intersection timescale. The intersection information (y-axis) is computed as a function of the time resolution (x-axis) and the encoding-readout intersection timescale is selected as the longest timescale (coarsest resolution) that gives the maximal intersection information. In this cartoon we show a case of an encoding timescale of 2 ms and an intersection timescale of 4 ms. This means that although sensory information is encoded at timescales as fine as 2 ms, only information encoded up to 4 ms is used to inform behavioral choices

One of the fundamental questions in neuroscience is determining what the neural code is. It is well known that neural signals come in the form of trains of action potentials, but we do not know what the unit of information is. A key question that has been often asked is the distinction between rate coding and spike timing coding. Neural responses can be measured by simply counting the number of spikes in a certain window or by measuring the timing of the spikes with a certain temporal resolution (Fig. 1A). Is the neural code just based (as in rate coding) on the number of spikes produced over some meaningful time window [e.g., the time it takes to perceive a stimulus (Thorpe et al. 1996)], or (as in spike timing coding) is the neural code made of the timing of individual spikes or by the temporal structure of patterns of spikes (Fellous et al. 2004; Victor 2000; Panzeri et al. 2010)?

The publication in 1995 of Mainen and Sejnowski’s (Mainen and Sejnowski 1995) seminal paper on reliability of spike timing renewed the interest, previously sparked by foundational theoretical work (MacKay and McCulloch 1952) and foundational experimental work (Bryant and Segundo 1976; Gray et al. 1989; Optican and Richmond 1987; Bialek et al. 1991), in how the timing of action potentials (which we term spike timing for brevity) contributes to the neural code. In the three decades since then, considerable experimental and theoretical research has investigated the timescales at which spike timing contributes to the neural code.

Characterizing these timescales of the neural code is important for several reasons. For experimental work, it tells us which timescales we should use to compute spike train features. For example, they tell us at which timescales we need to quantify neural activity for decoding it (Jacobs et al. 2009), or for documenting meaningful changes in neural activity across experimental conditions of interest. For neural network modelling or theoretical work, these timescales tell us which features of spike trains are essential for performing neural computations and thus should be well captured by models (Maass 2014; Maass and Markram 2004; Thorpe et al. 2001; Rullen and Thorpe 2001; Saddler and McDermott 2024; Chen et al. 2024; Akolkar et al. 2015).

**Fig. 2:**
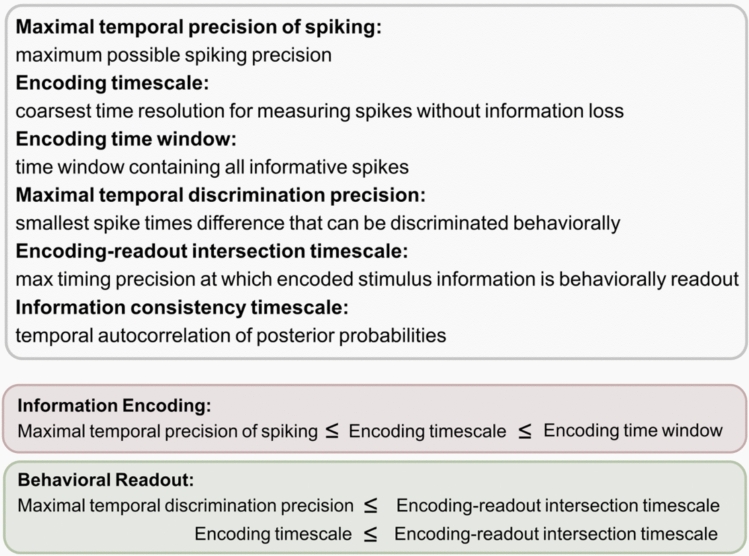
Different relevant spike timing timescales. Top: Glossary of the six timescales introduced in this paper. Bottom: Constraints on different timescales for information encoding and behavioral readout

Because of their importance for systems neuroscience, much effort has gone into understanding how to define and measure different types of timescales that correspond to different information processing functions. In this Review article, extending and updating previous reviews on the topic (Victor 2000; Panzeri et al. 2010, 2023; Cariani and Baker 2025), we synthetize work from the last 30 years to define, operationalize and measure these timescales. Through a critical review of the literature, we identify six broad classes of timescales (summarized in Fig. 2) that have been used in the literature of spike timing, and we describe how these timescales can be conceptualized and operationalized. We name these timescales as the maximal temporal precision of spiking that a neuron can achieve, the encoding time window (the time window containing all information-bearing spike times), the encoding timescale (the coarsest time resolution for measuring spikes without losing information), the maximal discrimination precision timescale (the smallest spike time difference that can be discriminated behaviorally), the encoding-readout intersection timescale (the maximal timing precision at which stimulus information encoded in neural activity is also actually read out to inform behavior), and the information consistency timescale (measuring the stability of information encoding over time).

We also attempt to distill the main messages and lessons that have been learned about brain function from the empirical measurements of these timescales across brain regions and experimental conditions. We argue that, together, this effort has revealed short and long timescales that have a profound influence on information coding and a major effect on behavior. For example, short encoding timescales, from millisecond to tens of milliseconds timescales, are useful for sensory information encoding and perception. Long consistency timescales, ranging from hundreds of milliseconds to seconds, are useful for accumulating evidence and stabilizing perceptual decisions.

## Maximal temporal precision of spiking

A first important timescale is the maximal temporal precision of spiking that a neuron can achieve. This timescale can be operationally estimated by using a repeatable input to the neuron (for example, a rapidly varying, highly controlled input current) and measuring the jitter of the spike times across repetitions of an identical stimulation. Although early neuroscience work assumed the spiking generation process to be very noisy and unable to reach to a high temporal precision in its spike timing (Adrian 1954), the work of Bryant and Segundo (1976) and of Mainen and Sejnowski (1995) has shown that this is not the case. On the contrary, neurons can have very high precision. When driving a cortical neuron to fire through stimulation with a controlled highly varying input current, high temporal precision of spiking on a millisecond scale can be achieved (Mainen and Sejnowski 1995).

The maximal timing precision is computed as the highest precision that can be obtained across experimental conditions with repeatable inputs. This may require investigating different types of inputs and selecting the ones that give the highest precision. For example, Mainen and Sejnowski found that spike times were highly repeatable, with timing reproducible at the millisecond timescale, when using a rapidly varying input current with fluctuations resembling those of in vivo synaptic activity, but were highly variable when using a constant (i.e. time-invariant) input current. In that case, the maximal precision of spiking is the millisecond-scale precision obtained from the repeatable spikes emitted in response to the fast-varying input current.

Because of the way it is defined, the maximal precision of spiking is computed without regard to what information the spike timing carries, for example without regard to whether the neuron under in vivo operating conditions carries information about e.g., visual or auditory stimuli. The maximal temporal precision sets an upper limit on the timing precision that a neuron can employ for processing information about specific variables, as described below.

## Encoding time window

Other timescales of information processing that have been considered are instead relative to the information carried by neural activity about specific cognitive variables of interest, such as the sensory stimuli that the subject is experiencing, the spatial position and so on. For simplicity, we call these variables “stimuli”, and we consider timescales relative to information about these stimuli.

The first important neural code timescale for information processing of specific features is the encoding time window (Theunissen and Miller 1995). As the name suggests, this window is related to one specific information processing operation: the encoding of information about the stimuli. We refer to encoding as the operation by which information about the stimuli is represented in single-trial neural activity. Experimentally, studying encoding of information about specific stimuli requires presenting many repetitions of a set of different stimuli (for example stimuli that differ in the value of a sensory feature such as the contrast or orientation of an image), and computing a measure of how well we can decode the presented stimulus feature from a single-trial observation of the spike times. This can be done using measures such as the mutual information (Shannon 1948) between stimuli and spike times, or the fraction of correct stimulus decoding across all trials (Quian Quiroga and Panzeri 2009). We refer colloquially to these measures as “information” between stimulus and spike times, without necessarily meaning that this measure relies on Shannon information theory. Importantly, measuring information requires both measuring variability at fixed stimulus and variability across stimuli (de Ruyter van Steveninck et al. 1997). High information needs low variability at fixed stimulus (that is, high spiking precision at fixed stimulus) and high variability across stimuli so that many stimuli can be represented by variations in the spike times.

**Fig. 3:**
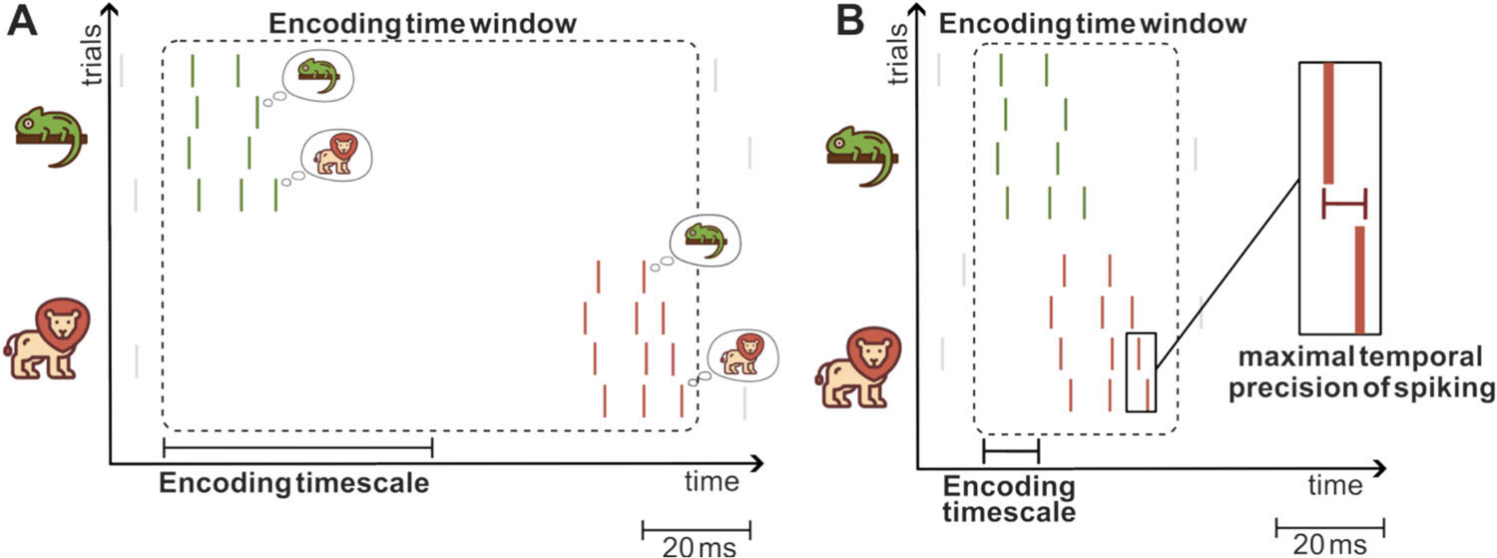
Schematic representation of spike timing coding and of some timescales of interest. **A** Cartoon of a spike raster of a hypothetical neuron responding to two different visual stimuli (a lion or a chameleon). Spikes emitted in response to the presentation of one of the two stimuli are color-coded (red: lion; green: chameleon). Each row corresponds to a different trial. The dashed-line box indicates the encoding time window, starting just after the stimulus presentation time (the origin of the x-axis) and ending when the spikes informative for stimulus discrimination stop being emitted. Here the maximal temporal precision of spiking is very short (a few ms, as spike times are highly repeatable across presentations of the same stimulus) but the encoding precision is long (~ 50 ms) because the set of spike times distinguishing the two stimuli is separated by ~ 50 ms. In this example we also assume that the readout mechanism for decision making is based on spike count during the entire encoding time window (chameleon is perceived for 2 spikes or less, and lion for 3 spikes or more, see speech bubbles). **B** A similar cartoon for a case where maximal precision of spike timing is short but the encoding precision is also short (because the two sets of spike times to the different stimuli are partly overlapping, a fine precision of approx. 10 ms is needed to gain information about which stimulus was presented). Animal icons from svgrepo.com, licensed under CC0

The encoding window is defined as the neural response window containing all particular response patterns that are considered as the code’s basic elements (Fig. 3A). This window is a fundamental parameter defining a code, but it is not known a priori. It has to be operationally determined with the following considerations (Theunissen and Miller 1995; Panzeri et al. 2010). For stimuli characterized by a single timescale, the length of the encoding window can be estimated empirically by considering periods in which the stimulus feature is constant and determining the shortest window that carries the entire information about the stimulus provided by the neuron (Fig. 1B). In the case of dynamic stimuli, this window must also be shorter than the timescales on which the relevant stimulus features change. For stimuli containing many timescales of variation, the above considerations apply focusing on the faster stimulus timescales (Theunissen and Miller 1995). As we will see below, this window is essential to measure other timescales considered below, and also to set the nature of encoding (e.g. to determine whether the temporal encoding is genuine).

The encoding time windows have been measured over different neural systems. They vary from very short, of the order of few tens of ms [for example, in rat somatosensory cortex encoding time windows are of the order of 30–40 ms for stimulus position (Panzeri et al. 2001)] to 100 ms or longer [for example, for encoding of static visual images in visual cortices (Victor 2000)] to even longer ones of several hundreds of ms [e.g. for gustatory responses (Di Lorenzo et al. 2009)].

## Encoding timescale

The timing of spikes can be used to encode information about sensory stimuli. Indeed, in many studies it has been shown that averaging the time course of neural activity over time, for example when computing time-averaged spike rates, leads to a considerable loss of information about stimuli encoded in both single neurons and populations, with respect to the amount of information that is encoded in the spike times measured with high temporal precision (Victor 2000; Panzeri et al. 2010). Therefore, a prominent feature of neural activity that shapes how and how much information is encoded in population activity is the encoding timescale, which defines the temporal precision at which information is represented in the timing of spikes (Tiesinga et al. 2008; Panzeri et al. 2010).

The temporal precision of the code can be operationally defined as the coarsest temporal resolution (that is, the longest timescale) at which spike times need to be measured without losing any information encoded in spike times with finer temporal precision (Panzeri et al. 2010; Theunissen and Miller 1995). Experimentally, this requires presenting different sensory stimuli, measuring the associated neural responses (Fig. 3A, B), and computing the amount of information about the stimuli that is encoded in the spike timing as a function of the temporal resolution used to measure the spikes (Victor 2000; Victor and Purpura 1996; Panzeri et al. 2001, 2010; Kayser et al. 2010; de Ruyter van Steveninck et al. 1997). The encoding timescale is the longest timescale (or coarsest temporal resolution) at which information about the stimuli extracted from neural activity stops increasing when increasing the precision (Fig. 1C). This quantity sets a fundamental timescale used for information representations in neural activity, because all decoders (e.g. downstream neural systems reading out this code or brain-machine interface algorithms), must be able to compute and operate with that temporal precision to extract all available neural information.

Extensive studies have characterized the encoding timescale. Often these studies have been performed using naturalistic stimuli (e.g. natural sounds when considering auditory stimuli or natural movies when considering visual stimuli), giving some degree of ecological validity to the encoding precisions that were found. These studies have found that the encoding timescale depends systematically on the hierarchical level of the brain area considered, on the type of sensory modality, and on the type of stimulus.

The encoding timescale is usually higher in peripheral and subcortical systems than in sensory cortices. Peripheral and thalamic neurons encode information with high precision (short timescales), ranging from finer than 1 ms to a few milliseconds (Jones et al. 2004; Montemurro et al. 2007; Moiseff and Konishi 1981; Gerstner et al. 1996; Butts et al. 2007; Arabzadeh et al. 2005), while cortical sensory neurons encode information at longer timescales, ranging from a few to a hundred milliseconds (Arabzadeh et al. 2005, 2006; Victor 2000; Schnupp et al. 2006; Kayser et al. 2010).

The encoding timescale also depends on the sensory modality (Table 1). At the primary sensory cortical level, the encoding timescale ranges from 2 to 5 ms for the somatosensory system (Arabzadeh et al. 2005, 2006; Panzeri et al. 2001; Long et al. 2022; Foffani et al. 2004, 2009; Stüttgen and Schwarz 2010) to 5–10 ms for the auditory system (Schnupp et al. 2006; Kayser et al. 2010; Engineer et al. 2008) to tens to hundreds of ms for the olfactory, visual and gustatory systems (Di Lorenzo et al. 2009; Victor 2000). The encoding timescale is also influenced by the stimulus variation timescales, with encoding precision being a few times faster than the fastest temporal scale of the stimulus (Butts et al. 2007; Kayser et al. 2010), analogous to what happens with efficient digital sampling of time-varying signals, in which several samples of the signal are taken in the time period of typical stimulus variations.

**Table 1:** Encoding timescales across primary sensory cortices (see main text)

Modality	Encoding timescale (ms)
Somatosensory	2–5
Auditory	5–10
Visual	10–100
Olfactory	10–100
Gustatory	> 100

Although the encoding timescale sets the finest temporal precision needed to recover all encoded information, this does not mean that this timescale is the only one at which information is encoded. It has often been reported that cortical neurons may multiplex sensory information (Panzeri et al. 2010; Kayser et al. 2009; Perez et al. 2013; Harvey et al. 2013; Victor 2000; Jagadisan and Gandhi 2022). That is, neurons use different timescales to simultaneously encode different types of information, for example different features of sensory stimuli. In such a case, a different encoding precision may be used for each stimulus variable encoded with time multiplexing.

Importantly, the relationship between the encoding time window and the encoding timescale sets whether the encoding of information is truly temporal (Theunissen and Miller 1995; Panzeri et al. 2010). The encoding of information in spike timing is defined truly temporal when the encoding time window is larger than the encoding timescale. In that case, the timing of spikes within the encoding window truly matters for information encoding, and the information in spike timing cannot simply follow the stimulus time variations. An example of genuine temporal encoding is encoding of stimulus location in the primary somatosensory cortex (Panzeri et al. 2001). In this study, we considered the encoding of a static (i.e. non-temporal) sensory variable, the location of the stimulated whisker. Primary somatosensory neurons encoded information within an encoding time window of 30–40 ms and with an encoding timescale of 5 ms. This means that the encoding of this static stimulus feature is truly temporal. Interestingly, in this study the encoding time window is short enough that typically up to one spike per neuron is emitted in response to whisker stimulation. This also implies that what matters for the encoding of stimulus location is by and large the timing of the first spike (a latency code), which has been postulated as computationally advantageous by theoretical studies (Thorpe et al. 2001; Rullen and Thorpe 2001).

The encoding timescale is expected to be longer than or equal to the maximal spiking precision timescale. In other words, the maximal spiking precision timescale bounds the timescales that are actually used to encode information about specific stimuli and particularly by naturalistic stimuli. The reason is that the encoding timescale cannot be finer than the precision of spike timing at fixed stimulus. If for example, the trial-averaged separation between the responses of two stimuli is long (100 ms) compared to the precision of spiking at fixed stimulus (5 ms), then measuring spikes with a resolution of 50 ms would be sufficient to decode the stimuli perfectly (Fig. 3A). However, if (as in Fig. 3B) the mean separation between the trial-averaged spike timing is shorter (say 10 ms so that the two spike timing distributions largely overlap), then the spikes will need to be read out with the precision close to the maximal precision to decode the stimuli. For obvious reasons, the maximal precision of spiking is higher or equal to the precision of spiking to individual stimuli, and thus ultimately the maximal precision of spiking bounds the encoding precision timescale.

## Maximal temporal discrimination precision

The fact that there is information about some stimuli encoded at a given timescale does not mean that this information is read out to perform behavioral discrimination between these stimuli (Softky 1995; Stevens and Zador 1995). Suppose for example that information is encoded by primary sensory afferents with a 1 ms encoding timescale, but that cortical neurons are sluggish in integrating spike times. In that case, information may be encoded peripherally with fine precision but not be usable for behavioral discrimination based on cortical computations.

**Fig. 4:**
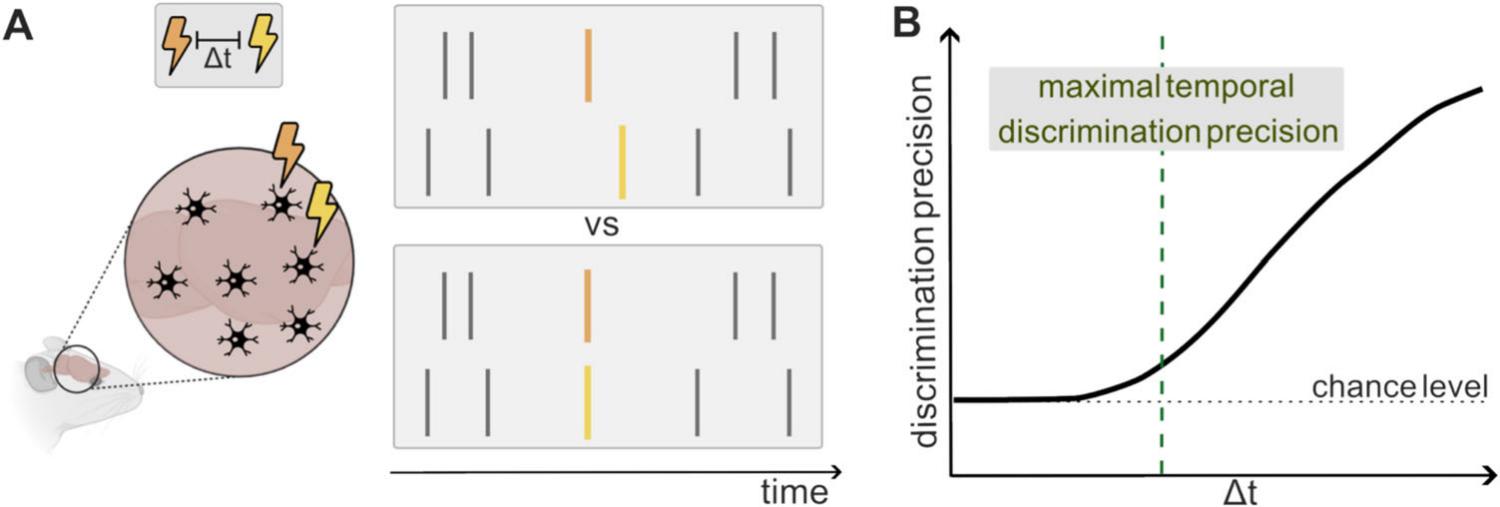
Maximal discrimination precision timescale. **A** Schematic illustration of the maximal discrimination precision timescale. It can be operationally measured with experiments using perturbations of neural activity (such as electrical microsimulation or optogenetics) to create responses in different neurons or populations separated by a certain interval Δt and by training the animal to discriminate these elicited patterns from synchronously elicited patterns from the same neurons or populations. The panels on the right show example spike trains: yellow and orange lines are stimulation-evoked spikes and gray lines are background spikes. **B** The smallest interval that can be discriminated significantly above chance defines the maximal temporal discrimination precision

One way to address whether spike times emitted with a certain precision can be decoded downstream to generate behavior is to evaluate the maximal temporal discrimination precision, defined as the minimal time difference between the activity of a neuron or neural population that can be discriminated behaviorally. It can be operationally measured with experiments using perturbations of neural activity such as electrical microsimulation (Houweling and Brecht 2008; Yang et al. 2008) or optogenetics (Histed and Maunsell 2014; Chong et al. 2020; Gill et al. 2020). These experiments are designed to create responses in different neurons or populations separated by a certain interval and by training the animal to discriminate these elicited patterns from synchronously elicited patterns in the same neurons or populations (Fig. 4A). The smallest interval that can be discriminated significantly measures the maximal temporal discrimination precision (Fig. 4B). This measure sets upper limits on the temporal precisions that can be exploited by the nervous system to produce behavioral discriminations by using spike timing. The maximal temporal discrimination precision gives complementary information with respect to the encoding timescale. If for example, a population of neurons encodes information about a certain sensory feature with encoding timescales as fine as 1 ms, but the maximal temporal discrimination precision measured from the same population is 10 ms, then only the information encoded up to 10 ms timescales is truly available for downstream computations. In this case sensory information encoded at scales finer than 10 ms down to the encoding timescale would be lost downstream and not used for behavior.

In a seminal series of studies using the above operational definition to measure the maximal temporal discrimination precision, rats were trained to discriminate between simultaneous and slightly offset electrical stimulation of two nearby sites in sensory cortex. Rats discriminated above chance inter-stimulation intervals as short as 1 ms, 3 ms and 15 ms when the stimulation electrodes were placed in somatosensory, auditory and visual cortex respectively (Yang et al. 2008; Yang and Zador 2012). These studies demonstrate that downstream neural structures can read out information encoded in cortical activity with high temporal precision. Interestingly, the maximal temporal discrimination precision of primary cortical neurons in each sensory modality matched well the corresponding encoding timescale precision of cortical neurons in the same modality that arise from studying information encoding of sensory stimuli (see previous section). This suggests that the stimulus information encoded in highly precise spike times during naturalistic stimuli in each sensory modality can, in principle, be read out. How to determine whether it is actually read out is discussed next.

## Encoding-readout intersection timescale

As we noted above, having information about stimuli encoded in spike timing at a very fine timescales does not mean that this information is actually read out when making perceptual judgments. While the above experiments measuring the maximal temporal discrimination precision can set upper bounds to how fine temporal discrimination between spike times could be, they do not tell which timescales are actually used when making discriminations about specific stimuli. For example, a system may have a high maximal temporal discrimination timescale of 1 ms. This precision may be used for example when discriminating very fine somatosensory textures whose difference is encoded by spike times at this fine timescale, but not used for discrimination when using coarse somatosensory textures even if the system is able to read out spike times, because coarse differences are robustly encoded by spike times measured over tens of ms (Arabzadeh et al. 2005, 2006).

A meaningful measure of the timescale at which information encoded about stimuli is read out to make perceptual discriminations must intersect the timescales at which information is encoded and those that can be read out. We call this the encoding-readout intersection timescale. How can we operationalize a measure of this timescale? We have recently proposed a definition of encoding-readout intersection based on the concept of intersection information (Panzeri et al. 2017). This concept is based on the idea that if a specific feature of neural activity (e.g. a first-spike latency measured with 1 ms precision) is used to inform a perceptual discrimination, then there should be a relationship between the quality of information encoded about the stimulus identity by the considered neural feature in a trial and the probability of making a correct perceptual judgement in the same trial (for example, if the feature gives faithful information about the stimulus in one trial, that is the stimulus can be correctly decoded from the feature in that trial, the probability of correct perceptual discrimination, or choice, should be higher in the considered trial than in trials in which the stimulus is not faithfully represented, for example if it gives incorrect decoding of the stimulus). This can be determined by quantifying the strength of the tri-variate relationships between presented stimulus, neural activity and the perceptual discrimination outcome. One way, based on information theory, is to compute how much of the stimulus information encoded in neural activity is reflected into the behavioral choice (Pica et al. 2017). Another way to measure this, based on linear decoders, is to construct a stimulus decoder of neural population activity and then compute how well the stimulus decoded from neural activity aligns on a trial-by-trial basis with the behavioral choice. This can be done, for example, by computing the fraction of trials in which both the stimulus is correctly decoded and the choice is correct (Panzeri et al. 2017), or by computing the correlation between the decoded stimulus and the choice (Park et al. 2025). The encoding-readout intersection timescale can be determined by measuring intersection information as function of the timing resolution used to measure spikes and identify it as the coarsest timing resolution at which the intersection information saturates (Fig. 1D).

A simpler approach to this question, that works at the trial-averaged level and does not track the trial-to-trial fluctuations between encoding and readout, is to compare the psychometric behavioral performance of the animal. For example, one could compare the fraction of correct discriminations to different stimuli with the stimulus discriminability obtained by decoding single-trial spike times (Luna et al. 2005; Engineer et al. 2008). With this measure, the encoding-readout intersection timescale is determined as the timescale for which the psychometric curve computed with spikes measured at this timescale maximizes the correlation with the psychometric curves (Engineer et al. 2008).

To illustrate cases in which encoding-readout intersection timescales may differ from the encoding timescale, we plotted (Fig. 3A) a cartoon of a hypothetical scenario in which information is encoded in spike timing, with a very high maximal precision of few ms and an intermediate encoding precision of ~ 50 ms (spikes to stimulus 1 are emitted with approximately 10 ms latency and with small trial-to-trial jitter, with mostly but not always 2 spikes per trial; spikes to stimulus 2 are emitted with approximately 90 ms latency and small trial-to-trial jitter, with mostly but not always 3 spikes per trial). If the readout only utilizes the total number of spikes emitted in the ~ 100 ms encoding window to decide whether stimulus 1 or 2 was presented, the encoding-readout intersection timescale would be of the size of the encoding window (100 ms) which is longer than the encoding timescale.

The encoding-readout timescale cannot be shorter than the encoding timescale (if no information is encoded down to a certain fine timescale, no information can be read out at that timescale to discriminate behaviorally between the stimuli). The encoding-readout intersection timescale coincides with the encoding timescale if the nervous system was optimal. In such a case, all encoded information would be read out, and there would be no distinction between encoding and readout. However, several recent studies have suggested that information readout may often be suboptimal. This has been shown e.g. by reporting that the discrimination of sensory stimuli from decoding neural activity is more accurate than behavioral performance (Stringer et al. 2021), or by demonstrating that the axes in neural information space that optimally decode choice are different from those that optimally decode the stimuli (Ni et al. 2018; Zhao et al. 2020; Valente et al. 2021). Because of possible suboptimalities, in general the encoding-readout timescale is expected to be larger than the encoding timescale when some information encoded at a fine timescale may not be read out (Figs. 1D, 3A), and equal to the encoding timescale when the encoding and readout timescales are well matched.

A few studies have measured the encoding-readout intersection timescale at which single neurons encode information. A study reported that the trial-averaged decoding performance obtained from auditory-cortex population spike times computed with 10 ms precision correlated better with trial-averaged behavioral discrimination than did decoding performance computed from population spike counts computed with poor temporal resolution (Engineer et al. 2008). Similar results were obtained in Callier et al. (2024), Stüttgen and Schwarz (2010) with a comparable approach applied to somatosensory coding in the somatosensory cortex. Another study used intersection information to link the information content about somatosensory textures of millisecond-precise spike timing on each trial to the behavioral discrimination of the texture made by the animal on the same trial. This study (Zuo et al. 2015; Pica et al. 2017) reported that in trials in which somatosensory cortical neurons were encoding faithfully the somatosensory stimuli, the behavioral performance in discriminating the same stimuli was better than in trials in which the same neurons were not encoding the stimuli faithfully. Thus, the amount of information encoded in spike times by the neurons correlated with the ability of the animal to correctly perform a perceptual judgment in the same trial. Because of this, millisecond-precise spike times had a high level of intersection information. In contrast, the information in spike counts computed with a coarser resolution of tens of milliseconds had a much lower relationship with the correctness of the perceptual discrimination and had much lower amounts of intersection information. However, another study in somatosensory cortex has found a better correlation with behavioral discrimination performance when decoding spike rates computed on coarse timescales over several tens of ms than with neural activity decoded using millisecond-precise spike timing (Luna et al. 2005). Together, these studies suggest that the information about sensory stimuli encoded in millisecond-scale activity can be read out to produce behavioral discriminations, and that readout timescales are often well matched with the encoding timescales. However, they also suggest that this is not always the case, and thus it is important to measure both encoding and encoding-readout alignment timescales.

That spike times are well read out and propagated by the nervous system may be surprising if we take the view that propagating precise spike timing downstream is difficult (Shadlen and Newsome 1998). We suggest that one reason that may contribute to precise spike timing favoring both encoding and readout is that precisely synchronizing spikes times of different neurons may enhance downstream signal propagations, for example when readout neurons have short time constants and act as coincidence detectors. This has been suggested by both foundational (Salinas and Sejnowski 2001; Koch et al. 1996; Tiesinga et al. 2008; Diesmann et al. 1999) and more recent (Valente et al. 2021; Ibáñez-Berganza et al. 2025) theoretical work. It has also been demonstrated by experiments using optogenetics to induce spatio-temporal patterns of activation in the olfactory bulb, showing that it is easier for a mouse to detect activation of small populations if performed with small (~ 10 ms) temporal jitters between the stimulated neurons (Gill et al. 2020). It is also compatible with recent work showing that correlations between the activity of different neurons are stronger in trials of perceptual discrimination tasks in which the animal made correct perceptual judgements (Valente et al. 2021; Francis et al. 2022; Balaguer-Ballester et al. 2020; Shahidi et al. 2019; Safaai et al. 2025).

## Information consistency timescale

**Fig. 5:**
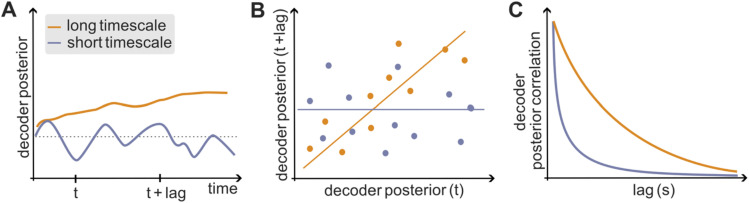
Information consistency timescale. **A** Longer consistency timescales mean that the time course of the instantaneous posterior probability of stimuli given the population vectors (obtained e.g. with a stimulus decoder based on population activity) is less variable across time. **B**, **C** The information consistency timescale can be operationalized by measuring the correlation across trials of the posterior probability at each time lag (**B**) and computing the decay time constant of this correlation as a function of the time lag (**C**)

A final timescale of importance in neural coding is the timescale of temporal consistency of encoding. This information consistency timescale captures the stability of information encoding over time. It can be operationalized (Fig. 5) as the correlation across time of the instantaneous stimulus encoding signal, quantified for example as the autocorrelation over time of the posterior probability of stimuli given the observation of a specific pattern of population activity at each specific time (Runyan et al. 2017). The information consistency timescale is fundamentally distinct from the encoding precision timescale. A fine encoding precision timescale (meaning that the instantaneous information in neural activity is encoded with high temporal precision) can coexist with either a long information consistency timescale (when the same fine-temporal-precision instantaneous information is repeated consistently in the same form over longer periods of time) or with a short information consistency timescale (when the fine-temporal-precision instantaneous information changes rapidly from time to time).

It has been argued that the existence of multiple neural temporal consistency timescales is crucial to produce complex computations (Runyan et al. 2017; Iigaya et al. 2019). For example, encoding rapidly changing sensory stimuli requires that information remains consistent only over a short timescale, of the order of the short timescales of stimulus variations. Accumulating evidence expressed at different timepoints to produce behavioral choices (Morcos and Harvey 2016; Akrami et al. 2018), or maintaining choice or goal signals consistently for periods long enough to implement goal-directed navigation plans (Runyan et al. 2017) may instead require maintaining consistent information over seconds.

The impact on behavior of having long temporal consistency timescales has been examined in a study that compared the information consistency timescale in PPC when a mouse performed an auditory discrimination task correctly or incorrectly (Runyan et al. 2017). The information consistency timescales generated by time-lagged correlated activity were longer in behaviorally correct trials than in behaviorally incorrect trials. This suggests that long timescales may be important for conveying signals crucial for accurate behavior. In the same study, no difference in consistency timescales was found between behaviorally correct and incorrect trials when recording neurons in auditory cortex rather than in Posterior Parietal Cortex (Runyan et al. 2017). This suggests that the benefits of long consistency timescales may be relevant specifically for higher-order association areas that have to integrate information over time and to guide decisions. This view is supported by recent experiments comparing population codes recorded from different areas during the same perceptual discrimination task. These studies reported that the timescales of population codes differ across the cortical hierarchy in a way that supports this hypothesis. During an auditory perceptual discrimination task, primary sensory areas had a much shorter information consistency timescale than the association area Posterior Parietal Cortex (Runyan et al. 2017). Comparable findings of longer information consistency timescales in association or decision areas, with respect to sensory areas, have been reported in tactile discrimination experiments (Fassihi et al. 2017). Also, findings of increases of information consistency timescales along the cortical hierarchy have been reported in the visual system when considering naturalistic movie stimulation (Piasini et al. 2021).

## Discussion

In this Review article, we systematically reviewed and conceptualized six different timescales that define spike timing that have been used across studies in the last 30 years. We hope that this will help readers to more easily elaborate the large body of work that has been done in measuring timescales that characterize the nature of spike trains and their contribution to brain computations and behavior.

Although initial studies on neural coding in the twentieth century mostly regarded spike timing as noisy and concentrated on averaging neural activity over long windows (spike rate coding) as a way to reduce this neural noise (Adrian 1954; Shadlen and Newsome 1998), a different picture has emerged since, as illustrated by the analysis of timescales reviewed here. In many systems, neurons can fire very precise spike timing (fine maximal precision of spiking). Precise spike timing is used to encode information, including information about naturalistic stimuli (short encoding timescales). Downstream systems have the capability to propagate fine timing information (short maximal precision discrimination timescale) and indeed seem to use this information to generate behaviors such as perceptual judgements (short encoding-readout intersection timescales often matching those of encoding). Moreover, the notion that spike timing codes are inherently more susceptible to noise has been challenged by findings that information encoded in spike timing is more stable to sensory noise (Kayser et al. 2009) or temporal drifts of representations (Zhu et al. 2025) than information encoded in spike counts. Thus, precise spike timing is an important part of the neural code meant as a unit of information used in the entire processing chain from emission to encoding to generation of behavior. This result, established over decades of careful work, was not necessarily expected at the beginning of this research. For example, several studies have objected to the behavioral relevance of latency codes on the argument that downstream decoders cannot read out a latency code because reading it out needs a representation of stimulus time. Other studies have challenged this view by showing that latency codes can be read out by relative timing (Stüttgen and Schwarz 2010; Panzeri et al. 2014) and that they indeed account for behavioral performance better than spike times do (Zuo et al. 2015).

More recent studies have also highlighted the importance of long timescales of consistency of information representations, of the order of hundreds of milliseconds to seconds to perform cognitive functions such as integration or accumulation of evidence for decision making or goal-directed spatial navigation (Runyan et al. 2017; Morcos and Harvey 2016; Akrami et al. 2018). These recent studies highlight that creating a diversity of timescales that implement different functions may be crucial to the operations of neural networks (Murray et al. 2014).

A limitation of the work reviewed here is that most, though not all, of the discussed evidence comes from observational studies of spike times during behavior. Although some studies have ameliorated some of these limitations by accounting for statistical correlations between information encoded in spike timing and behavior (Luna et al. 2005; Engineer et al. 2008; Jacobs et al. 2009; Zuo et al. 2015), to probe more causally the role of spike timing, precise manipulations of spike timing need to be designed and their effect on behavior tested (Adesnik and Abdeladim 2021; Panzeri et al. 2017). These experiments are very challenging because often nearby neurons may have different temporal response profiles to the stimuli (Zuo et al. 2015). This implies that stimulation applied without single cell resolution without knowing the temporal response profiles of each cell in response to the sensory stimuli would manipulate information in unpredictable ways (Panzeri et al. 2017; Adesnik and Abdeladim 2021). Alternative approaches may include identifying and intervening on the circuits that control spike timing and then assessing the effects that decreasing the ability of neural circuits to produce accurate spike timing has on behavior. As an example of this approach, a study using cell-type-specific optogenetic manipulations, found that layer V pyramidal cells control the encoding timescale of whisker deflection times in the primary somatosensory cortex through the activation of a specific inhibitory circuit, and that Layer V activity impacts behavioral reaction time in a whisker-based texture discrimination task (Vecchia et al. 2020). These findings also raise the important question of how different cell types, such as different classes of inhibitory neurons participate in the control of encoding and readout timescales (Wehr and Zador 2003; Cardin 2018; Rankin et al. 2024; Levi et al. 2022).

## Data Availability

No datasets were generated or analysed during the current study.
